# DNMT3A and DNMT3B in Breast Tumorigenesis and Potential Therapy

**DOI:** 10.3389/fcell.2022.916725

**Published:** 2022-05-10

**Authors:** Xiaxia Man, Qi Li, Baogang Wang, He Zhang, Songling Zhang, Ziyi Li

**Affiliations:** ^1^ Department of Oncologic Gynecology, the First Hospital of Jilin University, Jilin, China; ^2^ State and Local Joint Engineering Laboratory for Animal Models of Human Diseases, Academy of Translational Medicine, the First Hospital of Jilin University, Jilin, China; ^3^ Department of Cardiac Surgery, the First Hospital of Jilin University, Jilin, China

**Keywords:** methylation, Dnmt3a, DNMT3B, breast cancer, inhibitors

## Abstract

Breast cancer has become a leading cause of cancer-related deaths in women worldwide. DNA methylation has been revealed to play an enormously important role in the development and progression of breast cancer. DNA methylation is regulated by DNA methyltransferases (DNMTs), including DNMT1, DNMT2, and DNMT3. DNMT3 family has three members: DNMT3A, DNMT3B, and DNMT3L. The roles and functions of DNMT1 in breast cancer have been well reviewed. In this article, the roles of DNMT3A and DNMT3B in breast tumorigenesis and development are reviewed. We also discuss the SNP and mutations of DNMT3A and DNMT3B in breast cancer. In addition, we summarize how DNMT3A and DNMT3B are regulated by non-coding RNAs and signaling pathways in breast cancer, and targeting the expression levels of DNMT3A and DNMT3B may be a promising therapeutic approach for breast cancer. This review will provide reference for further studies on the biological functions and molecular mechanisms of DNMT3A and DNMT3B in breast cancer.

## Introduction

Breast cancer is a common malignant tumor among women and leads to cancer-related mortality in the world (Siegel et al., 2022). In 2020, female breast cancer has surpassed lung cancer as the most commonly diagnosed cancer with an estimated 2.3 million new cases (11.7%) followed by lung (11.4%) (Sung et al., 2021). Breast cancer is a highly heterogeneous disease that includes multiple intrinsic subtypes with heterogeneous molecular profiles, clinical representations, response to therapies and outcomes (Kerr et al., 2022). Although intensive chemotherapy, radiotherapy and targeted therapies have improved the outcomes of breast cancer patients, it is the fifth leading cause of cancer mortality worldwide, with 685,000 deaths (Sung et al., 2021).

**FIGURE 1:**
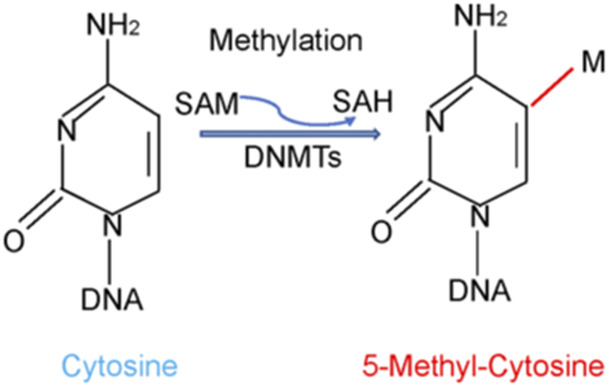
DNA methylation is regulated by DNMTs. DNA methylation is often regulated by DNA methyltransferases (DNMTs), which catalyzed methyl group from S-adenyl methionine (SAM) to C-5 position of the cytosine residue.

In general, DNA methylation is often regulated by DNMTs, which catalyzed methyl group attach to C-5 of the cytosine residue (Figure 1). Three canonical isoforms have been identified in human, including DNMT1, DNMT3A and DNMT3B (Liang et al., 2020; Zhu et al., 2021). Two non-canonical members are DNMT2 and DNMT3L. Among DNMTs isoforms, human tissues often express DNMT1, DNMT3A and DNMT3B isoforms (Liang et al., 2018; Hegde and Joshi, 2021). DNMT1 plays a critical role in maintenance methylation, while DNMT3A and DNMT3B function in *de novo* methylation to transfer a methyl group from S-adenyl methionine (SAM) to the C-5 position of cytosine residue (Okano et al., 1999; Lyko, 2018). However, several studies have confirmed that DNMT3A and DNMT3B also can maintain DNA methylation (Dodge et al., 2005; Feng et al., 2010). DNMT3C was identified in the male germ line and protected these germ cells from transposon activity (Barau et al., 2016).

Mounting evidence has indicated that DNA hypomethylation or hypermethylation and chromatin remodeling are critically involved in breast cancer development and malignant progression (Hinshelwood and Clark, 2008; Teschendorff et al., 2016; Pasculli et al., 2018). It has been reported that breast cancer patients have paradoxical gene-specific regional hypermethylation and global hypomethylation of the genome (Steeg et al., 2003). Regional hypermethylation leads to silence multiple genes involved in cell cycle and proliferation, while hypomethylation is required for tumor metastasis (Steeg et al., 2003). One group discovered the various roles of DNA methylation in different regulatory regions, which give rise to different breast cancer phenotypes (Fleischer et al., 2017). Higher expression of DNMT1 was displayed in the metastatic stage tissue samples, while higher expression of DNMT3A and DNMT3B was primarily exhibited in the primary stage, indicating that the expression of various DNMTs is tissue stage-dependent manner (Kar et al., 2014). Accumulated evidence suggests that DNMT3A and DNMT3B are pivotal in breast oncogenesis and progression.

Therefore, in this review, we described the latest findings of DNMT3A and DNMT3B in breast carcinogenesis, including their expression and clinical features, single-nucleotide polymorphisms (SNP), regulatory mechanisms, their biological functions. We also highlighted that targeting DNMT3A and DNMT3B could be useful for anti-breast cancer treatment. Hypomethylating agents and experimental DNMT inhibitors in breast cancer were also discussed.

### Expression and Clinical Features of DNMT3A and DNMT3B

Emerging evidence has showed that the expression of DNMT3A and DNMT3B is linked to clinical features in breast cancer patients. The expression of DNMT3A is higher in mammary tumors than in fibroadenoma (Yu et al., 2015). One group also reported that DNMT3A was highly expressed in breast tumor tissues than the adjacent normal specimens (Liu et al., 2016). In addition, DNMT3A was discovered to be highly expressed in breast cancer with brain metastases in comparison with primary breast cancer patients (Iwamoto et al., 2019). Breast cancer patients with advanced clinical stages often have high expression of DNMT3A and DNMT3B. Moreover, DNMT3A was correlated with a shorter DFS and OS in breast cancer patients (Yu et al., 2015). Another group indicated that higher expression of DNMT3A was observed in Grade III group and larger tumors in breast cancer patients (Kankava et al., 2016). Similarly, the mRNA levels of DNMT3A and DNMT3B were increased in tumor tissues compared with control groups (Jahangiri et al., 2018). These findings indicated that DNMT3A expression is linked to poor prognosis in breast cancer patients.

Interestingly, one study reported that DNMT3B expression was elevated in breast tumor subjects, but DNMT3A expression was not changed in breast cancer specimens in comparison to normal tissues, suggesting that deep exploration is necessary to determine the role of DNMT3A in breast cancer (Tavakolian et al., 2019). Another study reported that DNMT3B was highly expressed and predicted reduced survival in breast cancer patients (Shinden et al., 2021). Moreover, high expression of DNMT3B at mRNA level might be associated with lymph node diagnosis for breast cancer patients (Berger et al., 2006). By an array-based DNA methylation profiling, breast cancer patients with high methylation levels and upregulation of DNMT3B could have poor prognosis (Van der Auwera et al., 2010). Using differential high resolution melting analysis, one study revealed that DNMT3B promoter methylation was linked to cancer type, tumor size, histologic grade, suggesting that DNMT3B promoting methylation could predict diagnostic and prognostic biomarker for breast cancer (Naghitorabi et al., 2013). Therefore, DNMT3B overexpression is correlated with worse prognosis of breast cancer patients.

### Role of DNMT3A and DNMT3B in Breast Cancer

#### Downstream Targets of DNMT3A in Breast Cancer

DNMT3A plays its biological functions *via* regulating its downstream targets. One study showed that high expression of DNMT3A was linked to promoter hypermethylation of ERα and BRCA1, and downregulation of ERα and BRCA1 in breast cancer patients (Yu et al., 2015). Stable silencing of SOX2 oncoprotein via overexpression of DNMT3A in mice retarded the tumorigenic phenotype of breast cancer cells (Stolzenburg et al., 2015). DNMT3A can also enhance the methylation at non-CpGs and CpGs sites of HIF-1α in MDA-MB-231 cells (Li et al., 2019). Stilbenoid exposure resulted in SEMA 3A epigenetic activation via regulation of dynamic interactions of DNA with TF1C and DNMT3A in breast cancer cells (Beetch et al., 2019).

### Downstream Targets of DNMT3B in Breast Cancer

Accumulating evidence has uncovered multiple downstream targets of DNMT3B. DNMT3B overexpression is responsible for hypermethylation phenotype in multiple breast cancer cell lines (Roll et al., 2008). One group performed real-time RT-PCR and pointed out a three-gene expression signature, known as DNMT3B, BRCA2 and CCNE1, could be a useful prognostic marker (Bieche et al., 2004). Hypermethylation of WIF1 promoter was observed in 67% of breast cancer cells, which was mainly due to the cooperative activity of DNMT1 and DNMT3b, suggesting that WIF1 epigenetic silencing could link to Wnt dysregulation and breast carcinogenesis (Ai et al., 2006). Several studies have also indicated BRCA1 epigenetic inactivation caused poor survival was related with increased DNMT3B in sporadic breast cancers (Birgisdottir et al., 2006; Butcher and Rodenhiser, 2007; Chen et al., 2009).

One report showed that DNMT3B might service an accessory DNA methyltransferase to inhibit the expression of CXCL12 in MCF-7 breast cancer cells (Sowinska and Jagodzinski, 2007). Two ER-negative breast tumor cell lines, BCa-11 and BCa-15, were analyzed and found that these 2 cell lines had high expression of DNMT1 and DNMT3B transcript levels, and hypermethylation of several gene promoters, including HOXA5, RARβ2, and RASSF1A (Raju Bagadi et al., 2008). One group identified that DNMT3B, DNMT3L, DNMT1, EZH2, and Mecp2 controlled the non-CpG methylation of Notch-3 gene in TNBC cells (Xiao et al., 2020). DNMT3B targets GNB4 DNA methylation in breast cancer cells, contributing to downregulation of DNA methylation and suppression of proliferation of breast cancer cells (Wang et al., 2018). FOXM1 was found to bind to CRY2 promoter *via* interaction with DNMT3B, leading to downregulation of CRY2 (Liu et al., 2017). Demethylation agent 5-aza-dc, an inhibitor of DNMT3A and DNMT3B, increased the expression of caspase 8 and maspin in breast cancer cell lines (Wu et al., 2010). DNMT1, DNMT3A and DNMT3B increased the DNA methylation of FOXF2 in breast cancer cells (Tian et al., 2015). SP-1 can bind with FOXF2 promoter region and block the DNA methylation of FOXF2 in breast cancer cells, leading to promotion of cell proliferation in basal-like breast cancer (Tian et al., 2015). These studies identified the function of DNMT3B via regulating downstream targets in breast cancer.

### Single Nucleotide Polymorphisms (SNPs) of DNMT3A and DNMT3B

DNMT gene polymorphisms are associated with breast oncogenesis (Table 1). One study identified 16 SNPs in DNMTs, including 5 SNPs in DNMT1, 6 SNPs in DNMT3A, 3 SNPs in DNMT3B, 1 SNP in DNMT3L and 1 SNP in DNMT2 in 408 breast cancer patients and 469 controls. Moreover, the heterozygous genotypes of rs2424908 in DNMT3B was linked to decreased risk of breast cancer in Han Chinese women (Sun et al., 2012). In a British population, the C46359T polymorphism in the DNMT3B promoter in breast cancer cases was reported by investigation of 352 breast cancer patients and 258 controls, indicating that individuals with T allele in DNMT3B have a high risk of breast cancer development (Montgomery et al., 2004). Interestingly, there is no relationship between DNMT3B polymorphisms and the risk of breast cancer in Chinese women (Ye et al., 2010). It is necessary to mention that DNMT3A and DNMT3B have mutation and amplification in breast cancer patients. DNMT3B gene amplification was observed in breast cancer cells and was associated with resistance to DNA demethylating drugs, including Decitabine, 5-azacytidine (Vidaza), and SGI-1027 (Simo-Riudalbas et al., 2011). By a mutational analysis, 10% DNMT3A was found to have high frequent mutation in metastatic breast cancer patients (Rong et al., 2021).

**TABLE 1:** SNP of DNMT in breast cancer.

Item	DNMT1	DNMT2	DNMT3A	DNMT3B	DNMT3L	Ref
SNP	rs2114724, rs2228611, rs2228612, rs8101866, and rs16999593	rs11254413	rs13420827, rs11887120, rs13428812, rs1550117, rs11695471, and rs6733301	DNMT3A; rs2424908, rs2424913, and rs6087990	s113593938	Sun et al. (2012)
SNP				C46359T		(Montgomery et al., 2004; Eftekhar et al., 2014)
SNP	rs2228611		rs1550117, rs7581217	rs2424908		Terrazzino et al. (2017)
						

### Regulation of DNA Methylation in Breast Cancer

#### miRNAs Regulate DNA Methylation

**FIGURE 2:**
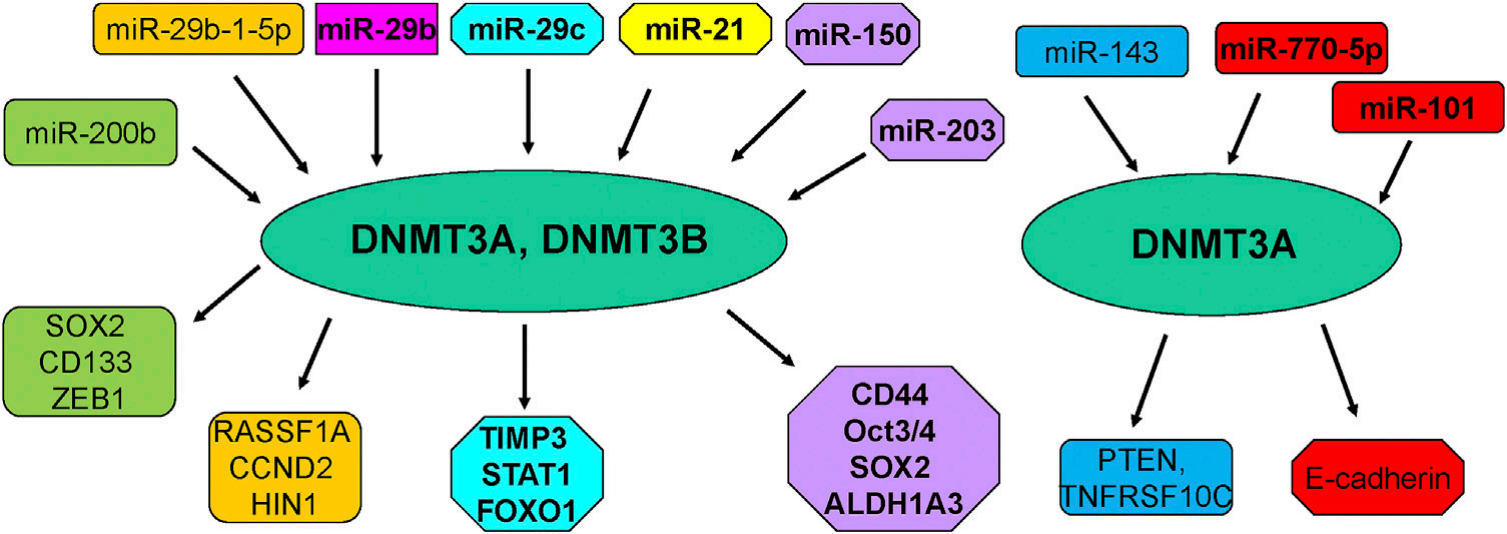
miRNAs regulate DNA methylation in breast cancer.

Several noncoding RNAs have been reported to affect DNA methylation in breast cancer cells (Pronina et al., 2017) (Table 2; Figure 2). One study analyzed the association between multiple miRNAs expression and DNMT3B-induced DNA hypermethylation, and concluded that dysregulation of miRNAs led to aberrant DNA hypermethylation via suppressing post-transcriptional level of DNMT3B in basal-like breast tumor (Sandhu et al., 2014). This group also reported several miRNAs such as miR-26b, miR-29c and miR-148b regulated the expression of DNMT3B in multiple breast cancer cells (Sandhu et al., 2012). Another group showed that microRNA-29b (miR-29b) can bind with 3′-UTR of DNMT3A and DNMT3B and inhibit the mRNA level of DNMT3A and DNMT3B, leading to multiple gene promoter methylation in breast cancer cells and suppression of cell proliferation (Starlard-Davenport et al., 2013). This study suggested that miR-29b could regulate DNMT3A and DNMT3B and suppress proliferation of breast cancer cells (Starlard-Davenport et al., 2013).

**TABLE 2:** miRNAs regulate DNMTs in breast cancer.

Noncoding RNAs	DNMTs	Targets	Functions	Ref
miR-29b	DNMT3A, DNMT3B	N/A	Suppresses proliferation	Starlard-Davenport et al. (2013)
miR-29b-1-5p	DNMT1, DNMT3A, DNMT3B	RASSF1A, CCND2, HIN1	Increases ROS generation, reduces cell proliferation	De Blasio et al. (2020)
miR-29c	DNMT3A, DNMT3B	TIMP3, STAT1, FOXO1	Reduces the proliferation, migration, and invasion	Li et al. (2018)
miR-143	DNMT3A	PTEN, TNFRSF10C	Suppresses proliferation	Ng et al. (2014)
miR-200b	DNMT3A, DNMT3B	SOX2, CD133, ZEB1	Performs antitumor activity	(Yu et al., 2013; Pang et al., 2018)
miR-101	DNMT3A	E-cadherin	Inhibits proliferation, migration	Liu et al. (2016)
miR-21	DNMT1, DNMT3A, DNMT3B	N/A	Promotes tumorigenesis	Zhang et al. (2015)
miR-150	DNMT3A, DNMT3B	CD44, Oct3/4, SOX2, ALDH1A3	*Cancer* stem cell development	El-Osaily et al. (2021)
miR-203	DNMT3A, DNMT3B	CD44, Oct3/4, SOX2, ALDH1A3	*Cancer* stem cell development	El-Osaily et al. (2021)
miR-770-5p	DNMT3A	E-cadherin	Inhibits EMT and invasion	Noyan et al. (2021)

Moreover, low expression of miR-29b was observed and negatively associated with the expression of DNMT3A in primary ER-positive breast cancer patients (Shinden et al., 2015). Similarly, overexpression of miR-29b-1-5p repressed the expression of DNMT1, DNMT3A and DNMT3B and elevated the expression of RASSF1A, CCND2 and HIN1 in breast cancer cells (De Blasio et al., 2020). In consistent, miR-29c-5p was revealed to negatively target the DNMT3A in ER-positive breast cancer (Aure et al., 2021). In line with this report, miR-29c inhibited the expression of DNMT3B and subsequently reduced the expression of TIMP3 and affected STAT1/FOXO1 signaling pathway in breast cancer cells (Li et al., 2018).

Another group clarified that miR-143 decreased the expression of DNMT3A at mRNA and protein levels, and subsequently decreased the PTEN hypermethylation and enhanced TNFRSF10C methylation, leading to suppression of proliferation of breast cancer cells (Ng et al., 2014). Similarly, miR-124a-3 hypermethylation was associated with high expression of DNMT3B and linked to aggressive and advanced stages in breast cancer patients (Ben Gacem et al., 2014). Kindlin 2, a focal adhesion protein to govern Wnt signaling pathway, can bind with DNMT3A and co-occupy the miR-200b promoter, leading to downregulation of miR-200b and promotion of invasion of breast cancer cells (Yu et al., 2013). In addition, another study revealed that MYC can recruit DNMT3A to bind with miR-200b promoter and caused CpG island hypermethylation, leading to miR-200b suppression in MDA-MB-231 cells and upregulation of SOX2, CD133 and ZEB1 (Pang et al., 2018). Interestingly, miR-200b also directly reduced the expression of DNMT3A expression in TNBC cells (Pang et al., 2018). Moreover, miR-200b, miR-200c and miR-221 target DNMT3B expression, while DNMT3B can also stimulate the DNA methylation of miR-200s in CAFs and further indicated that TGF-β1/miR-200s/miR-221/DNMT3B axis governed CAF status to stimulate proliferation of breast cancer cells (Tang et al., 2019).

In keeping with this finding, another investigation also confirmed miR-221 maintained CSCs via suppressing DNMT3B and increasing the expression of stemness genes in breast cancer, such as Nanog and Oct3/4 genes (Roscigno et al., 2016). FEN1 was reported to elevate the expression of DNMT3A and promote their interaction among FEN1/PCNA/DNMT3A and suppressed the expression of miR-200a-5p, leading to upregulation of miR-200a-5p targets, MET and EGFR, which contribute to enhancement of growth of breast cancer cells (Zeng et al., 2019). Overexpression of miR-101 caused the downregulation of DNMT3A and subsequent upregulation of E-cadherin expression in MDA-MB-231 cells, which is responsible for suppression of proliferation and migration capacity of breast cancer cells (Liu et al., 2016).

Additionally, inhibition of miR-21 expression led to the increased genome DNA methylation level and promotion of DNMT1, DNMT3A, DNMT3B expressions in MCF-7 cells, whereas upregulation of miR-21 decreased genome DNA methylation and inhibited DNMT3B expression in MDA-MB-231 cells (Zhang et al., 2015). Interestingly, 5-AZA treatment upregulated miR-21 expression in MCF-7 and MDA-MB-231 cells, suggesting that DNA methylation also affects the miR-21 expression in breast cancer cells (Zhang et al., 2015). Twist increased the expression of miR-22 via combination with DNMT3B and HDAC1, and repressed ERα in breast cancer cells, facilitating tamoxifen resistance (Vesuna et al., 2012; Vesuna et al., 2021). Besides, miR-770-5p repressed invasion and EMT via inhibition of DNMT3A and restoration of E-cadherin in MDA-MB-231 TNBC cells (Noyan et al., 2021). Moreover, miR-203 and miR-150 can regulate the expression of DNMT3A and DNMT3B in breast cancer cells, resulting in regulation of CD44, ALDH1A3, OCT3/4, SOX2 expression, which are critical for breast cancer stem cell development (El-Osaily et al., 2021). Clearly, numerous miRNAs regulate the expression of DNMT3A and DNMT3B in breast cancer.

### LncRNAs and piRNA Regulate DNA Methylation

LncRNA is a type of long noncoding RNA, which plays an essential role in diseases, including tumors (Liang et al., 2019; Jiang et al., 2020a; Liu and Shang, 2022). Piwi-interacting RNA (piRNA) is a class of small noncoding RNA, which can interact with argonaute proteins, leading to regulation of downstream targets (Gamez et al., 2020; Xu et al., 2021). There is evidence that lncRNAs and piRNAs can regulate DNA methylation in breast cancer (Table 3). For example, lncRNA 01638 downregulation repressed the expression of DNMT1, DNMT3A and DNMT3B, and increased the expression of PTEN and BRCA1, resulting in inhibition of proliferation and invasion in HER2-positive breast cancer cells (Liu et al., 2019). Similarly, lncRNA 00922 recruited three DNMTs in the NKD2 promoter to trigger NKD2 methylation and repress its expression, contributing to activation of Wnt signaling pathway and breast cancer progression (Wang et al., 2021). LncRNA MIAT bound to three DNMTs and increased DLG3 promoter methylation and suppressed DLG3 expression, which caused inactivation of the Hippo pathway and promotion of breast cancer progression (Li D. et al., 2020).

**TABLE 3:** LncRNAs and circRNAs regulate DNMTs in breast cancer.

Noncoding RNAs	DNMTs	Targets	Functions	Ref
Linc01638	DNMT1, DNMT3A, DNMT3B	PTEN, BRCA1	Inhibits proliferation, invasion	Liu et al. (2019)
Linc00922	DNMTs	NKD2, Wnt	Promotes tumor progression	Wang et al. (2021)
LncRNA MIAT	DNMTs	DLG3, Hippo	Promotes tumor progression	Li et al. (2020a)
MALAT1	DNMTs	BRCA1, PTEN	Regulates Herceptin resistance	Yang et al. (2021)
LncRNA H19	DNMT3B	Beclin 1	Decreases tamoxifen resistance	Wang et al. (2019)
CircIQCH	DNMT3A	miR-145	Facilitates tumor progression	Li et al. (2020c)
piR-823	DNMTs	APC, Wnt	Induces CSCs	Ding et al. (2021)

LncRNA MALAT1 increased the expression of DNMT1, DNMT3A and DNMT3B and inhibited the BRCA1 and PTEN expression, leading to regulating of herceptin sensitivity in HER2-positive breast cancer (Yang et al., 2021). LncRNA H19 depletion promoted the interaction between DNMT3B and Beclin 1 promoter, causing the Beclin 1 DNA methylation, decreasing tamoxifen resistance due to autophagy inhibition in breast cancer (Wang et al., 2019). Li and others identified that circRNA circIQCH (hsa_circ_0,104,345) facilitated breast cancer progression via sponging miR-145 and increasing DNMT3A expression (Li Y. et al., 2020). Recently, piR-823 increased the expression of DNMTs, including DNMT1, DNMT3A and DNMT3B, enhanced APC DNA methylation and subsequently activated Wnt pathway, leading to induction of CSCs in luminal breast cancer (Ding et al., 2021).

### Signaling Pathways Regulate DNA Methylation

Multiple signaling pathways are validated to govern DNA methylation in breast cancer and some of these pathways are related to DNMT3a/DNMT3b. FEN1-mediated DNMT3a up-regulation, released the suppression of its targeting MET and EGFR, and promoted breast cancer cell proliferation by activating PI3K/AKT and MAPK/ERK pathways in MCF-7cells (Zeng et al., 2019). In addition, Wang *et al.* showed that DNMT3a interacts with p53 signaling in maintaining genome and represses p53-mediated transactivation of the p21 gene (Wang et al., 2005). DNMT3B affected many signaling pathways, such as STAT3, PI3K/Akt, *β*-catenin, NF-κB and Notch pathways (So et al., 2020).

The Oxidative DNA Damage might also be related to DNMT3b. One study in the 2020 revealed a novel mechanism underlying the modulation of DNA methylation patterns induced by oxidative DNA damage at the tumor suppressor BRCA1 gene through the coordination between pol *β* and DNMT3b (Jiang Z. et al., 2020). Furthermore, one study determined a molecular mechanism by which DNMT3b7 (aberrant DNMT3b transcripts) promotes tumor progression in breast cancer cells through hypermethylation and loss of *CDH1/E-cadherin* expression, altered *β*-catenin localization, and subsequent changes in cell adhesion, proliferation, and growth in soft agar (Brambert et al., 2015). Therefore, targeting these signaling pathways could be helpful for regulation of DNA methylation in breast cancer.

### Targeting DNA Methylation in Breast Cancer

DNA methylation is a reversal process, suggesting that DNA methylation is a promising therapeutic target (Wu and Zhang, 2014; Pechalrieu et al., 2017). Several pre-clinical or clinical evidence has validated that tumor suppressor methylation is associated with prognosis of breast cancer patients (Stearns et al., 2007; Pouliot et al., 2015). Previous studies in our laboratory observed that 6-TG blocked DNMT1 activity markedly, leading to inhibition of MDA-MB-231 cell growth and induction of apoptosis through reactivating methylation-silenced genes in the apoptosis pathway and PI3K–AKT signaling pathways, and also inducing FAS-mediated exogenous apoptosis and p21-dependent G2/M arrest in MCF-7 breast cancer cells (Li H. et al., 2020; Zhang et al., 2020; Chu et al., 2022).

A variety of breast cancer-related preclinical studies indicated the anti-tumor potential for the nucleoside analogues azacitidine and decitabine. For instance, one study showed that protein levels of DNMTs were associated with response to decitabine in TNBC cells as examined in TNBC patient-derived xenograft organoids, and all three DNMTs (DNMT1, DNMT3A and DNMT3B) were degraded by decitabine treatment *in vitro* and *in vivo* (Yu et al., 2018). Several breast cancer-related clinical trials by DNMT inhibitors (NCT01349959, NCT00978250, NCT03295552, NCT00748553) are going. Although some patients may respond to these DNMT inhibitors, in most patients they are ineffective in part due to drug administration, drug distribution and selection of DNMT isoforms. Using DNMT inhibitors still faces enormous challenges. We believe that targeting DNMTs remains an attractive approach for the development of novel therapies for breast cancer patients.

## Conclusion and Prospects

In conclusion, DNMT3A and DNMT3B play an enormously critical role in the occurrence and development of breast cancer. There are several issues that need to be addressed regarding the role of DNMTs in breast cancer. For example, DNMTs target numerous genes for their DNA methylations. Which gene methylation is the key driver to trigger breast cancer development? All DNMT1, DNMT3A and DNMT3B are involved in breast carcinogenesis. Which DNMT is critical to participate in breast oncogenesis? Since noncoding RNAs and signaling pathways regulate the expression of DNMT3A and DNMT3B, targeting these noncoding RNAs and pathways is an alternative approach to control the DNMT3A and DNMT3B expression. Due to the critical role of DNMTs in breast tumorigenesis, targeting DNMTs might be a potential approach for breast cancer therapy. Many compounds are validated to target several DNMTs in breast cancer cells. It is better to discover the potentially specific inhibitors for individual DNMT for breast cancer treatment. Moreover, whether these inhibitors of DNMTs can be used clinically for treating breast cancer needs to be answered. Therefore, further investigation of roles of DNMTs in breast tumorigenesis will help us to design novel therapeutic strategy *via* targeting DNMTs for breast cancer.

## Abbreviations

ABC, ATP binding cassette; AhR, aromatic hydrocarbon receptor; APC, adenomatous polyposis *coli;* APL, acute promyelocytic leukemia; 5-aza-dc, 5-aza-2′-deoxycytidine; BER, base excision repair; BRCA1, breast cancer susceptibility gene 1; CAF, cancer-associated fibroblasts; COMT, Catechol-O-methyltransferase; CSCs, cancer stem cells; DNMT3; DNA methyltransferase 3; FOXF2, forkhead box F2; H3K9me3, trimethylated H3K9; GNB4, guanine nucleotide-binding protein beta-4; IGFBP-3, insulin-growth factor binding protein-3; Linc01638, long intergenic non-coding RNA 01638; LINE-1, long interspersed nuclear element-1; MAML1, Mastermind-like transcriptional coactivator 1; MBD2, methyl binding protein 2; MIAT, myocardial infarction associated transcript; PiRNAs, PIWI-interacting small non-coding RNAs; SEMA 3A, semaphoring 3A; SNPs, single nucleotide polymorphisms; SOX2, sex determining region Y-box 2; TDG, Thymine DNA glycosylase; WIF1, Wnt inhibitory factor-1.

## References

[B1] Ai L. Tao Q. Zhong S. Fields C. R. Kim W.-J. Lee M. W. (2006). Inactivation of Wnt Inhibitory Factor-1 (WIF1) Expression by Epigenetic Silencing Is a Common Event in Breast Cancer. Carcinogenesis 27, 1341–1348. 10.1093/carcin/bgi379 16501252

[B2] Aure M. R. Fleischer T. Fleischer T. Bjørklund S. Ankill J. Castro-Mondragon J. E. (2021). Crosstalk between microRNA Expression and DNA Methylation Drives the Hormone-dependent Phenotype of Breast Cancer. Genome Med. 13, 72. 10.1186/s13073-021-00880-4 33926515 PMC8086068

[B3] Bagadi S. A. R. Kaur J. Ralhan R. (2008). Establishment and Characterisation of Two Novel Breast Cancer Cell Lines. Cell. Biol. Int. 32, 55–65. 10.1016/j.cellbi.2007.08.010 17959394

[B4] Barau J. Teissandier A. Zamudio N. Roy S. Nalesso V. Hérault Y. (2016). The DNA Methyltransferase DNMT3C Protects Male Germ Cells from Transposon Activity. Science 354, 909–912. 10.1126/science.aah5143 27856912

[B5] Beetch M. Lubecka K. Shen K. Flower K. Harandi-Zadeh S. Suderman M. (2019). Stilbenoid-Mediated Epigenetic Activation of Semaphorin 3A in Breast Cancer Cells Involves Changes in Dynamic Interactions of DNA with DNMT3A and NF1C Transcription Factor. Mol. Nutr. Food Res. 63, e1801386. 10.1002/mnfr.201801386 31327173

[B6] Ben Gacem R. Ben Abdelkrim O. Ziadi S. Ben Dhiab M. Trimeche M. (2014). Methylation of miR-124a-1, miR-124a-2, and miR-124a-3 Genes Correlates with Aggressive and Advanced Breast Cancer Disease. Tumor Biol. 35, 4047–4056. 10.1007/s13277-013-1530-4 24375250

[B7] Berger J. Mueller-Holzner E. Fiegl H. Marth C. Daxenbichler G. (2006). Evaluation of Three mRNA Markers for the Detection of Lymph Node Metastases. Anticancer Res. 26, 3855–3860. 17094413

[B8] Bièche I. Tozlu S. Girault I. Lidereau R. (2004). Identification of a Three-Gene Expression Signature of Poor-Prognosis Breast Carcinoma. Mol. Cancer 3, 37. 10.1186/1476-4598-3-37 15606925 PMC544833

[B9] Birgisdottir V. Stefansson O. A. Bodvarsdottir S. K. Hilmarsdottir H. Jonasson J. G. Eyfjord J. E. (2006). Epigenetic Silencing and Deletion of the BRCA1gene in Sporadic Breast Cancer. Breast Cancer Res. 8, R38. 10.1186/bcr1522 16846527 PMC1779478

[B10] Blasio A. Di Fiore R. Pratelli G. Drago‐Ferrante R. Saliba C. Baldacchino S. (2020). A Loop Involving NRF2, miR‐29b‐1‐5p and AKT, Regulates Cell Fate of MDA‐MB‐231 Triple‐negative Breast Cancer Cells. J. Cell. Physiology 235, 629–637. 10.1002/jcp.29062 31313842

[B11] Brambert P. R. Kelpsch D. J. Hameed R. Desai C. V. Calafiore G. Godley L. A. (2015). DNMT3B7 expression promotes tumor progression to a more aggressive phenotype in breast cancer cells. *PLoS One* 10, e0117310. 10.1371/journal.pone.0117310 PMC4301645 25607950

[B12] Butcher D. T. Rodenhiser D. I. (2007). Epigenetic Inactivation of BRCA1 Is Associated with Aberrant Expression of CTCF and DNA Methyltransferase (DNMT3B) in Some Sporadic Breast Tumours. Eur. J. Cancer 43, 210–219. 10.1016/j.ejca.2006.09.002 17071074

[B13] Chen Y. Zhou J. Xu Y. Li Z. Wen X. Yao L. (2009). BRCA1promoter Methylation Associated with Poor Survival in Chinese Patients with Sporadic Breast Cancer. Cancer Sci. 100, 1663–1667. 10.1111/j.1349-7006.2009.01225.x 19522853 PMC11158407

[B14] Chu M. An X. Zhang D. Li Q. Dai X. Yu H. (2022). Combination of the 6-thioguanine and disulfiram/Cu Synergistically Inhibits Proliferation of Triple-Negative Breast Cancer Cells by Enhancing DNA Damage and Disrupting DNA Damage Checkpoint. Biochimica Biophysica Acta (BBA) - Mol. Cell. Res. 1869, 119169. 10.1016/j.bbamcr.2021.119169 34763028

[B15] Ding X. Li Y. Lü J. Zhao Q. Guo Y. Lu Z. (2021). piRNA-823 Is Involved in Cancer Stem Cell Regulation through Altering DNA Methylation in Association with Luminal Breast Cancer. Front. Cell. Dev. Biol. 9, 641052. 10.3389/fcell.2021.641052 33791297 PMC8005588

[B16] Dodge J. E. Okano M. Dick F. Tsujimoto N. Chen T. Wang S. (2005). Inactivation of Dnmt3b in Mouse Embryonic Fibroblasts Results in DNA Hypomethylation, Chromosomal Instability, and Spontaneous Immortalization. J. Biol. Chem. 280, 17986–17991. 10.1074/jbc.m413246200 15757890

[B17] Eftekhar E. Rasti M. Nahgibalhossaini F. Sadeghi Y. (2014). The Study of DNA Methyltransferase-3B Promoter Variant Genotype Among Iranian Sporadic Breast Cancer Patients. Iran. J. Med. Sci. 39, 268–274. 24850984 PMC4027006

[B18] El-Osaily H. H. Ibrahim I. H. Essawi M. L. Salem S. M. (2021). Impact of miRNAs Expression Modulation on the Methylation Status of Breast Cancer Stem Cell-Related Genes. Clin. Transl. Oncol. 23, 1440–1451. 10.1007/s12094-020-02542-0 33433838

[B19] Feng J. Zhou Y. Campbell S. L. Le T. Li E. Sweatt J. D. (2010). Dnmt1 and Dnmt3a Maintain DNA Methylation and Regulate Synaptic Function in Adult Forebrain Neurons. Nat. Neurosci. 13, 423–430. 10.1038/nn.2514 20228804 PMC3060772

[B20] Fleischer T. Tekpli X. Tekpli X. Mathelier A. Wang S. Nebdal D. (2017). DNA Methylation at Enhancers Identifies Distinct Breast Cancer Lineages. Nat. Commun. 8, 1379. 10.1038/s41467-017-00510-x 29123100 PMC5680222

[B21] Gamez S. Srivastav S. Akbari O. S. Lau N. C. (2020). Diverse Defenses: A Perspective Comparing Dipteran Piwi-piRNA Pathways. Cells 9(10):2180. 10.3390/cells9102180 PMC7601171 32992598

[B22] Hegde M. Joshi M. B. (2021). Comprehensive Analysis of Regulation of DNA Methyltransferase Isoforms in Human Breast Tumors. J. Cancer Res. Clin. Oncol. 147, 937–971. 10.1007/s00432-021-03519-4 33604794 PMC7954751

[B23] Hinshelwood R. A. Clark S. J. (2008). Breast Cancer Epigenetics: Normal Human Mammary Epithelial Cells as a Model System. J. Mol. Med. 86, 1315–1328. 10.1007/s00109-008-0386-3 18716754

[B24] Iwamoto T. Niikura N. Ogiya R. Yasojima H. Watanabe K.-i. Kanbayashi C. (2019). Distinct Gene Expression Profiles between Primary Breast Cancers and Brain Metastases from Pair-Matched Samples. Sci. Rep. 9, 13343. 10.1038/s41598-019-50099-y 31527824 PMC6746866

[B25] Jahangiri R. Mosaffa F. Emami Razavi A. Teimoori‐Toolabi L. Jamialahmadi K. (2018). Altered DNA Methyltransferases Promoter Methylation and mRNA Expression Are Associated with Tamoxifen Response in Breast Tumors. J. Cell. Physiol. 233, 7305–7319. 10.1002/jcp.26562 29574992 PMC13482140

[B26] Jiang W. Xia J. Xie S. Zou R. Pan S. Wang Z.-w. (2020a). Long Non-coding RNAs as a Determinant of Cancer Drug Resistance: Towards the Overcoming of Chemoresistance via Modulation of lncRNAs. Drug Resist. Updat. 50, 100683. 10.1016/j.drup.2020.100683 32146422

[B27] Jiang Z. Lai Y. Beaver J. M. Tsegay P. S. Zhao M. L. Horton J. K. (2020b). Gene via the Crosstalk between DNA Polymerase Beta and a De Novo DNA Methyltransferase. *Cells* 9. Oxidative DNA Damage Modul. DNA Methylation Pattern Hum. Breast Cancer 1 (BRCA1). 10.3390/cells9010225 PMC7016758 31963223

[B28] Kankava K. Kvaratskhelia E. Abzianidze E. (2016). A Study of the Relationship between Levels of Methyltransferases in Peripheral Blood Mononuclear Cells and Characteristics of Tumor in Patients with Ductal Invasive Carcinoma of Breast. Georgian Med. News, 31–35. 27845283

[B29] Kar S. Sengupta D. Deb M. Shilpi A. Parbin S. Rath S. K. (2014). Expression Profiling of DNA Methylation-Mediated Epigenetic Gene-Silencing Factors in Breast Cancer. Clin. Epigenet 6, 20. 10.1186/1868-7083-6-20 PMC4255691 25478034

[B30] Kerr A. J. Dodwell D. Mcgale P. Holt F. Duane F. Mannu G. (2022). Adjuvant and Neoadjuvant Breast Cancer Treatments: A Systematic Review of Their Effects on Mortality. Cancer Treat. Rev. 105, 102375. 10.1016/j.ctrv.2022.102375 35367784 PMC9096622

[B31] Li C. Xiong W. Liu X. Xiao W. Guo Y. Tan J. (2019). Hypomethylation at Non-CpG/CpG Sites in the Promoter of HIF-1α Gene Combined with Enhanced H3K9Ac Modification Contribute to Maintain Higher HIF-1α Expression in Breast Cancer. Oncogenesis 8, 26. 10.1038/s41389-019-0135-1 30940798 PMC6445832

[B32] Li D. Hu X. Yu S. Deng S. Yan M. Sun F. (2020a). Silence of lncRNA MIAT-Mediated Inhibition of DLG3 Promoter Methylation Suppresses Breast Cancer Progression via the Hippo Signaling Pathway. Cell. Signal. 73, 109697. 10.1016/j.cellsig.2020.109697 32593652

[B33] Li H. An X. Zhang D. Li Q. Zhang N. Yu H. (2020b). Transcriptomics Analysis of the Tumor-Inhibitory Pathways of 6-Thioguanine in MCF-7 Cells via Silencing DNMT1 Activity. Ott Vol 13, 1211–1223. 10.2147/ott.s236543 PMC7023860 32103989

[B34] Li W. Yi J. Zheng X. Liu S. Fu W. Ren L. (2018). miR-29c Plays a Suppressive Role in Breast Cancer by Targeting the TIMP3/STAT1/FOXO1 Pathway. Clin. Epigenet 10, 64. 10.1186/s13148-018-0495-y PMC5956756 29796115

[B35] Li Y. Jiang B. He Z. Zhu H. He R. Fan S. (2020c). circIQCH Sponges miR-145 to Promote Breast Cancer Progression by Upregulating DNMT3A Expression. Aging 12, 15532–15545. 10.18632/aging.103746 32756009 PMC7467367

[B36] Liang J. Chen W. Lin J. (2019). LncRNA: An All-Rounder in Rheumatoid Arthritis. J. Transl. Int. Med. 7, 3–9. 10.2478/jtim-2019-0002 30997350 PMC6463828

[B37] Liang Z. Hu J. Yan W. Jiang H. Hu G. Luo C. (2018). Deciphering the Role of Dimer Interface in Intrinsic Dynamics and Allosteric Pathways Underlying the Functional Transformation of DNMT3A. Biochimica Biophysica Acta (BBA) - General Subj. 1862, 1667–1679. 10.1016/j.bbagen.2018.04.015 29674125

[B38] Liang Z. Zhu Y. Long J. Ye F. Hu G. (2020). Both intra and Inter-domain Interactions Define the Intrinsic Dynamics and Allosteric Mechanism in DNMT1s. Comput. Struct. Biotechnol. J. 18, 749–764. 10.1016/j.csbj.2020.03.016 32280430 PMC7132064

[B39] Liu J. Pang Y. Wang H. Li Y. Sun X. Xu F. (2016). miR-101 Inhibits the Proliferation and Migration of Breast Cancer Cells via Downregulating the Expression of DNA Methyltransferase 3a. Xi Bao Yu Fen Zi Mian Yi Xue Za Zhi 32, 299–303. 26927545

[B40] Liu J. Shang G. (2022). The Roles of Noncoding RNAs in the Development of Osteosarcoma Stem Cells and Potential Therapeutic Targets. Front. Cell. Dev. Biol. 10, 773038. 10.3389/fcell.2022.773038 35252166 PMC8888953

[B41] Liu L. Shen H. Wang Y. (2017). CRY2 Is Suppressed by FOXM1 Mediated Promoter Hypermethylation in Breast Cancer. Biochem. Biophysical Res. Commun. 490, 44–50. 10.1016/j.bbrc.2017.06.003 28579430

[B42] Liu P. Tang H. Wu J. Qiu X. Kong Y. Zhang L. (2019). Linc01638 Promotes Tumorigenesis in HER2+ Breast Cancer. Curr. Cancer Drug Targets 19, 74–80. 10.2174/1568009618666180709163718 29992881 PMC6327113

[B43] Lyko F. (2018). The DNA Methyltransferase Family: a Versatile Toolkit for Epigenetic Regulation. Nat. Rev. Genet. 19, 81–92. 10.1038/nrg.2017.80 29033456

[B44] Montgomery K. G. Liu M. C. Eccles D. M. Campbell I. G. (2004). The DNMT3B C→T Promoter Polymorphism and Risk of Breast Cancer in a British Population: a Case-Control Study. Breast Cancer Res. 6, R390–R394. 10.1186/bcr807 15217506 PMC468658

[B45] Naghitorabi M. Mohammadi Asl J. Mir Mohammad Sadeghi H. Rabbani M. Jafarian-Dehkordi A. Javanmard H. S. (2013). Quantitative Evaluation of DNMT3B Promoter Methylation in Breast Cancer Patients Using Differential High Resolution Melting Analysis. Res. Pharm. Sci. 8, 167–175. 24019826 PMC3764668

[B46] Ng E. K. O. Li R. Shin V. Y. Siu J. M. Ma E. S. K. Kwong A. (2014). MicroRNA-143 Is Downregulated in Breast Cancer and Regulates DNA Methyltransferases 3A in Breast Cancer Cells. Tumor Biol. 35, 2591–2598. 10.1007/s13277-013-1341-7 24218337

[B47] Noyan S. Andac Ozketen A. Gurdal H. Gur Dedeoglu B. (2021). miR-770-5p Regulates EMT and Invasion in TNBC Cells by Targeting DNMT3A. Cell. Signal. 83, 109996. 10.1016/j.cellsig.2021.109996 33798630

[B48] Okano M. Bell D. W. Haber D. A. Li E. (1999). DNA Methyltransferases Dnmt3a and Dnmt3b Are Essential for De Novo Methylation and Mammalian Development. Cell. 99, 247–257. 10.1016/s0092-8674(00)81656-6 10555141

[B49] Pang Y. Liu J. Li X. Xiao G. Wang H. Yang G. (2018). MYC and DNMT3A-Mediated DNA Methylation Represses microRNA-200b in Triple Negative Breast Cancer. J. Cell. Mol. Med. 22, 6262–6274. 10.1111/jcmm.13916 30324719 PMC6237581

[B50] Pasculli B. Barbano R. Parrella P. (2018). Epigenetics of Breast Cancer: Biology and Clinical Implication in the Era of Precision Medicine. Seminars Cancer Biol. 51, 22–35. 10.1016/j.semcancer.2018.01.007 29339244

[B51] Pechalrieu D. Etievant C. Arimondo P. B. (2017). DNA Methyltransferase Inhibitors in Cancer: From Pharmacology to Translational Studies. Biochem. Pharmacol. 129, 1–13. 10.1016/j.bcp.2016.12.004 27956110

[B52] Pouliot M. C. Labrie Y. Diorio C. Durocher F. (2015). The Role of Methylation in Breast Cancer Susceptibility and Treatment. Anticancer Res. 35, 4569–4574. 26254344

[B53] Pronina I. V. Loginov V. I. Burdennyy A. M. Fridman M. V. Senchenko V. N. Kazubskaya T. P. (2017). DNA Methylation Contributes to Deregulation of 12 Cancer-Associated microRNAs and Breast Cancer Progression. Gene 604, 1–8. 10.1016/j.gene.2016.12.018 27998789

[B54] Roll J. D. Rivenbark A. G. Jones W. D. Coleman W. B. (2008). DNMT3b Overexpression Contributes to a Hypermethylator Phenotype in Human Breast Cancer Cell Lines. Mol. Cancer 7, 15. 10.1186/1476-4598-7-15 18221536 PMC2246151

[B55] Rong G. Yi Z. Ma F. Guan Y. Xu Y. Li L. (2021). DNA Damage Response as a Prognostic Indicator in Metastatic Breast Cancer via Mutational Analysis. Ann. Transl. Med. 9, 220. 10.21037/atm-20-2137 33708847 PMC7940884

[B56] Roscigno G. Quintavalle C. Donnarumma E. Puoti I. Diaz-Lagares A. Iaboni M. (2016). MiR-221 Promotes Stemness of Breast Cancer Cells by Targeting DNMT3b. Oncotarget 7, 580–592. 10.18632/oncotarget.5979 26556862 PMC4808019

[B57] Sandhu R. Rivenbark A. G. Coleman W. B. (2012). Loss of Post-transcriptional Regulation of DNMT3b by microRNAs: a Possible Molecular Mechanism for the Hypermethylation Defect Observed in a Subset of Breast Cancer Cell Lines. Int. J. Oncol. 41, 721–732. 10.3892/ijo.2012.1505 22664488 PMC3982716

[B58] Sandhu R. Rivenbark A. G. Mackler R. M. Livasy C. A. Coleman W. B. (2014). Dysregulation of microRNA Expression Drives Aberrant DNA Hypermethylation in Basal-like Breast Cancer. Int. J. Oncol. 44, 563–572. 10.3892/ijo.2013.2197 24297604 PMC3898722

[B59] Shinden Y. Hirashima T. Nohata N. Toda H. Okada R. Asai S. (2021). Molecular Pathogenesis of Breast Cancer: Impact of miR-99a-5p and miR-99a-3p Regulation on Oncogenic Genes. J. Hum. Genet. 66, 519–534. 10.1038/s10038-020-00865-y 33177704

[B60] Shinden Y. Iguchi T. Akiyoshi S. Ueo H. Ueda M. Hirata H. (2015). miR-29b Is an Indicator of Prognosis in Breast Cancer Patients. Mol. Clin. Oncol. 3, 919–923. 10.3892/mco.2015.565 26171207 PMC4486821

[B61] Siegel R. L. Miller K. D. Fuchs H. E. Jemal A. (2022). Cancer Statistics, 2022. CA A Cancer J. Clin. 72, 7–33. 10.3322/caac.21708 35020204

[B62] Simó-Riudalbas L. Melo S. A. Esteller M. (2011). DNMT3B Gene Amplification Predicts Resistance to DNA Demethylating Drugs. Genes Chromosom. Cancer 50, 527–534. 10.1002/gcc.20877 21484930

[B63] So J. Y. Skrypek N. Yang H. H. Merchant A. S. Nelson G. W. Chen W.-D. (2020). Induction of DNMT3B by PGE2 and IL6 at Distant Metastatic Sites Promotes Epigenetic Modification and Breast Cancer Colonization. Cancer Res. 80, 2612–2627. 10.1158/0008-5472.can-19-3339 32265226 PMC7299749

[B64] Sowinska A. Jagodzinski P. P. (2007). RNA Interference-Mediated Knockdown of DNMT1 and DNMT3B Induces CXCL12 Expression in MCF-7 Breast Cancer and AsPC1 Pancreatic Carcinoma Cell Lines. Cancer Lett. 255, 153–159. 17532557 10.1016/j.canlet.2007.04.004

[B65] Starlard-Davenport A. Kutanzi K. Tryndyak V. Word B. Lyn-Cook B. (2013). Restoration of the Methylation Status of Hypermethylated Gene Promoters by microRNA-29b in Human Breast Cancer: A Novel Epigenetic Therapeutic Approach. J. Carcinog. 12, 15. 10.4103/1477-3163.115720 23961262 PMC3746452

[B66] Stearns V. Zhou Q. Davidson N. E. (2007). Epigenetic Regulation as a New Target for Breast Cancer Therapy. Cancer Investig. 25, 659–665. 10.1080/07357900701719234 18058459

[B67] Steeg P. S. Ouatas T. Halverson D. Palmieri D. Salerno M. (2003). Metastasis Suppressor Genes: Basic Biology and Potential Clinical Use. Clin. Breast Cancer 4, 51–62. 10.3816/cbc.2003.n.012 12744759

[B68] Stolzenburg S. Beltran A. S. Swift-Scanlan T. Rivenbark A. G. Rashwan R. Blancafort P. (2015). Stable Oncogenic Silencing *In Vivo* by Programmable and Targeted De Novo DNA Methylation in Breast Cancer. Oncogene 34, 5427–5435. 10.1038/onc.2014.470 25684141 PMC4633433

[B69] Sun M.-Y. Yang X.-X. Xu W.-W. Yao G.-Y. Pan H.-Z. Li M. (2012). Association of DNMT1 and DNMT3B Polymorphisms with Breast Cancer Risk in Han Chinese Women from South China. Genet. Mol. Res. 11, 4330–4341. 10.4238/2012.september.26.1 23079992

[B70] Sung H. Ferlay J. Siegel R. L. Laversanne M. Soerjomataram I. Jemal A. (2021). Global Cancer Statistics 2020: GLOBOCAN Estimates of Incidence and Mortality Worldwide for 36 Cancers in 185 Countries. CA A Cancer J. Clin. 71, 209–249. 10.3322/caac.21660 33538338

[B71] Tang X. Tu G. Yang G. Wang X. Kang L. Yang L. (2019). Autocrine TGF-β1/miR-200s/miR-221/dnmt3b Regulatory Loop Maintains CAF Status to Fuel Breast Cancer Cell Proliferation. Cancer Lett. 452, 79–89. 10.1016/j.canlet.2019.02.044 30851420 PMC7560952

[B72] Tavakolian S. Goudarzi H. Faghihloo E. (2019). E-cadherin, Snail, ZEB-1, DNMT1, DNMT3A and DNMT3B Expression in Normal and Breast Cancer Tissues. Acta Biochim. Pol. 66, 409–414. 10.18388/abp.2019_2808 31880901

[B73] Terrazzino S. Deantonio L. Cargnin S. Donis L. Pisani C. Masini L. (2017). DNA Methyltransferase Gene Polymorphisms for Prediction of Radiation-Induced Skin Fibrosis after Treatment of Breast Cancer: A Multifactorial Genetic Approach. Cancer Res. Treat. 49, 464–472. 10.4143/crt.2016.256 27554481 PMC5398398

[B74] Teschendorff A. E. Gao Y. Jones A. Ruebner M. Beckmann M. W. Wachter D. L. (2016). DNA Methylation Outliers in Normal Breast Tissue Identify Field Defects that Are Enriched in Cancer. Nat. Commun. 7, 10478. 10.1038/ncomms10478 26823093 PMC4740178

[B75] Tian H.-P. Lun S.-M. Huang H.-J. He R. Kong P.-Z. Wang Q.-S. (2015). DNA Methylation Affects the SP1-Regulated Transcription of FOXF2 in Breast Cancer Cells. J. Biol. Chem. 290, 19173–19183. 10.1074/jbc.m114.636126 26070560 PMC4521039

[B76] Van Der Auwera I. Yu W. Suo L. Van Neste L. Van Dam P. Van Marck E. A. (2010). Array-based DNA Methylation Profiling for Breast Cancer Subtype Discrimination. PLoS One 5, e12616. 10.1371/journal.pone.0012616 20830311 PMC2935385

[B77] Vesuna F. Lisok A. Kimble B. Domek J. Kato Y. Van Der Groep P. (2012). Twist Contributes to Hormone Resistance in Breast Cancer by Downregulating Estrogen Receptor-α. Oncogene 31, 3223–3234. 10.1038/onc.2011.483 22056872 PMC3276743

[B78] Vesuna F. Lisok A. Van Diest P. Raman V. (2021). Twist Activates miR-22 to Suppress Estrogen Receptor Alpha in Breast Cancer. Mol. Cell. Biochem. 476, 2295–2306. 10.1007/s11010-021-04065-w 33582945 PMC9128495

[B79] Wang B. Li D. Rodriguez-Juarez R. Farfus A. Storozynsky Q. Malach M. (2018). A Suppressive Role of Guanine Nucleotide-Binding Protein Subunit Beta-4 Inhibited by DNA Methylation in the Growth of Anti-estrogen Resistant Breast Cancer Cells. BMC Cancer 18, 817. 10.1186/s12885-018-4711-0 30103729 PMC6090602

[B80] Wang J. Xie S. Yang J. Xiong H. Jia Y. Zhou Y. (2019). The Long Noncoding RNA H19 Promotes Tamoxifen Resistance in Breast Cancer via Autophagy. J. Hematol. Oncol. 12, 81. 10.1186/s13045-019-0747-0 31340867 PMC6657081

[B81] Wang Y. A. Kamarova Y. Shen K. C. Jiang Z. Hahn M. J. Wang Y. (2005). DNA Methyltransferase-3a Interacts with P53 and Represses P53-Mediated Gene Expression. Cancer Biol. Ther. 4, 1138–1143. 10.4161/cbt.4.10.2073 16131836

[B82] Wang Y. Dong T. Wang P. Li S. Wu G. Zhou J. (2021). LINC00922 Regulates Epithelial-Mesenchymal Transition, Invasive and Migratory Capacities in Breast Cancer through Promoting NKD2 Methylation. Cell. Signal. 77, 109808. 10.1016/j.cellsig.2020.109808 33045317

[B83] Wu H. Zhang Y. (2014). Reversing DNA Methylation: Mechanisms, Genomics, and Biological Functions. Cell. 156, 45–68. 10.1016/j.cell.2013.12.019 24439369 PMC3938284

[B84] Wu Y. Alvarez M. Slamon D. J. Koeffler P. Vadgama J. V. (2010). Caspase 8 and Maspin Are Downregulated in Breast Cancer Cells Due to CpG Site Promoter Methylation. BMC Cancer 10, 32. 10.1186/1471-2407-10-32 20132554 PMC2824712

[B85] Xiao W. Liu X. Niu X. Li C. Guo Y. Tan J. (2020). The Frequency of CpG and Non-CpG Methylation of Notch3 Gene Promoter Determines its Expression Levels in Breast Cancer Cells. Exp. Cell. Res. 386, 111743. 10.1016/j.yexcr.2019.111743 31770532

[B86] Xu Y. Song G. Xie S. Jiang W. Chen X. Chu M. (2021). The Roles of PD-1/pd-L1 in the Prognosis and Immunotherapy of Prostate Cancer. Mol. Ther. 29, 1958–1969. 10.1016/j.ymthe.2021.04.029 33932597 PMC8178461

[B87] Yang C. Zhu H. Tan Y. Zhu R. Wu X. Li Y. (2021). MALAT1 Promotes Tumorigenesis and Increases Cellular Sensitivity to Herceptin in HER2-Positive Breast Cancer. Curr. Cancer Drug Targets. 10.2174/1568009621666210618164300 34148540

[B88] Ye C. Beeghly-Fadiel A. Lu W. Long J. Shu X. O. Gao Y.-T. (2010). Two-stage Case-Control Study of DNMT-1 and DNMT-3B Gene Variants and Breast Cancer Risk. Breast Cancer Res. Treat. 121, 765–769. 10.1007/s10549-009-0569-9 19798569 PMC3493111

[B89] Yu J. Qin B. Moyer A. M. Nowsheen S. Liu T. Qin S. (2018). DNA Methyltransferase Expression in Triple-Negative Breast Cancer Predicts Sensitivity to Decitabine. J. Clin. Invest. 128, 2376–2388. 10.1172/jci97924 29708513 PMC5983332

[B90] Yu Y. Wu J. Guan L. Qi L. Tang Y. Ma B. (2013). Kindlin 2 Promotes Breast Cancer Invasion via Epigenetic Silencing of the microRNA200 Gene Family. Int. J. Cancer 133, 1368–1379. 10.1002/ijc.28151 23483548

[B91] Yu Z. Xiao Q. Zhao L. Ren J. Bai X. Sun M. (2015). DNA Methyltransferase 1/3a Overexpression in Sporadic Breast Cancer Is Associated with Reduced Expression of Estrogen Receptor-Alpha/breast Cancer Susceptibility Gene 1 and Poor Prognosis. Mol. Carcinog. 54, 707–719. 10.1002/mc.22133 24464625

[B92] Zeng X. Qu X. Zhao C. Xu L. Hou K. Liu Y. (2019). FEN1 Mediates miR‐200a Methylation and Promotes Breast Cancer Cell growthviaMET and EGFR Signaling. FASEB J. 33, 10717–10730. 10.1096/fj.201900273r 31266372

[B93] Zhang D. An X. Li Q. Man X. Chu M. Li H. (2020). Thioguanine Induces Apoptosis in Triple-Negative Breast Cancer by Regulating PI3K-AKT Pathway. Front. Oncol. 10, 524922. 10.3389/fonc.2020.524922 33194583 PMC7662440

[B94] Zhang Y. Y. Tian W. P. Mei M. (2015). Interaction between miR-21 and DNA Methylation in Different Breast Cancer Cells. Zhongguo Ying Yong Sheng Li Xue Za Zhi 31, 220–224. 26387181

[B95] Zhu M. Ding Q. Lin Z. Chen X. Chen S. Zhu Y. (2021). New Insights of Epigenetics in Vascular and Cellular Senescence. J. Transl. Int. Med. 9, 239–248. 10.2478/jtim-2021-0049 35136723 PMC8802399

